# Creating and moving nanoantenna cold spots anywhere

**DOI:** 10.1038/s41377-022-00893-7

**Published:** 2022-08-30

**Authors:** Alex J. Vernon, Francisco J. Rodríguez-Fortuño

**Affiliations:** Department of Physics, King’s College London, Strand, London, WC2R 2LS United Kingdom

**Keywords:** Nanophotonics and plasmonics, Nanophotonics and plasmonics, Optical techniques

## Abstract

Cold spots are sub-wavelength regions which might emerge near a nanoantenna, should one or more components of some far-field illumination cancel out with scattered light. We show that by changing only the polarisation, amplitude, and phase of two plane waves, a unique, zero-magnitude and highly sub-wavelength cold spot can be created and moved anywhere in the space around a nanoantenna of any arbitrary shape. This can be achieved using ultra-fast modulated pulses, or a time-harmonic approximation. Easily disturbed by a change in the nanoantenna’s material or position, a manufactured cold spot is fragile and could be used in nanoscale sensing. Our technique exploits the linearity of Maxwell’s equations and could be adapted to manipulate any phenomena governed by the linear wave equation, including acoustic scattering. This is a means for potentially ultra-fast sub-wavelength electric field manipulation.

## Introduction

If visible light shines on a small particle, power will be scattered in different directions. By changing the particle’s shape, much of this power can be squeezed into confined pockets of space, referred to as hot spots^1,2^. In exchange, dimmer regions develop around the particle where the scattered and incident light begin to cancel out, and any dark zone where one or more components of interest are completely suppressed is called a cold spot^3,4^.

Plasmonic nanoantennas are engineered to scatter optical-frequency light in a valuable way^5,6^, with many structures designed to brighten received light in hot spots by a factor of over 1000. Such highly intense hot spots have been applied to photovoltaics^7–12^, and are a natural asset to fluorescence^13,14^ and surface-enhanced Raman scattering spectroscopy^15–18^ as well as biosensing and other nanomedicine applications^19–23^. Meanwhile, advanced microscopy techniques have emerged in the last twenty years, relying on fluorescent molecules to beat the diffraction limit of light and produce super-resolution images. Fluorophores can be switched ‘on’ and ‘off’ by light, which is the basis for the two fundamental approaches used amongst PALM/STORM^24,25^, STED^26^ and MINFLUX^27^. While PALM/STORM stochastically activate molecules one by one to discover their relative positions, STED and MINFLUX localise molecules by shining with a beam containing electric field zeros; these zeros are commonly delivered in two-dimensional form by a doughnut beam and are similar to cold spots in the nanoantenna context. Hot and cold spots have already seen use as fluorophore switches, and in one example two quantum dots were selectively addressed by changing the handedness of some elliptically polarised incident light^28^. An alternative application for cold spots might exploit the fact that a beam, blue-detuned with respect to a certain electron transition, can push atoms into wells of low electric field intensity^29^. If an illuminated nanostructure unlocks the ability to manipulate an electric field of appropriate wavelength, atoms could be trapped and easily manoeuvred in controllable cold spots. Whether or not a hot or cold spot emerges in a nanoantenna’s near field depends on its geometry and material properties, and the incoming light. The phase shift introduced by the nanoantenna as it scatters light is tuneable by changing the antenna length, making it possible to enhance or practically eliminate one component of the overall electric field in one or more known locations^30^. Swappable hot and cold spots have also been demonstrated in the nanogap of two rods by changing only the polarisation of the incident field^31^. Similarly promising techniques offer ultra-fast hot spot switching between different locations near a nanostructure using Fourier limited or chirped pulses^32^, or by changing the direction of propagation of the incident light^33^. Unfortunately, hot spots are tightly bound to the shape of the nanoantenna and are not free to be moved anywhere in space. But a cold spot is. In this work, we show that with simple and potentially ultrafast changes in the polarisation of the illuminating light, the electric fields near a nanoantenna can be designed to destructively interfere in the right places and steer a cold spot along an arbitrary path in space. This way, a cold spot’s movement can be mapped to an incident field’s polarisation signature on the Poincaré sphere.

**Fig. 1:**
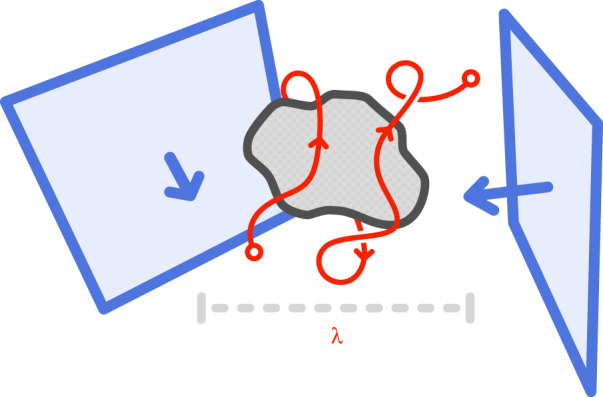
The concept A cold spot moved in an arbitrary path (red line) around a nanoparticle by the changing polarisation of two plane waves

The components of an incident field, such as a plane wave, set up different electric fields upon interacting with the nanoantenna. Balancing the share of power and the phase difference between these components, it is possible to control some aspects of the total field in the nanoantenna’s surroundings^34,35^. Each plane wave component affords a handle to turn and manipulate the electric field surrounding the antenna; given enough handles, we can create a cold spot wherever we like. The minimum number of controllable plane wave components needed incident on the antenna depends on how many components of the total electric field we wish to extinguish. Here, we present full position control of a zero-magnitude, three-dimensional cold spot by changing only the electric field components of two plane waves (Fig. 1), justified next. Though we apply our work to the context of electric fields around a nanoantenna, the underlying idea is valid for any phenomenon which follows the linear wave equation – including scalar acoustic fields, surface water waves, or even gravitational waves in linearised gravity, for example.

## Results

### Degrees of freedom

Lit by a single plane wave ${{{\mathbf{E}}}}_{{{\mathrm{a}}}}\left( {{{\mathbf{r}}}} \right){{{\mathrm{e}}}}^{ - i\omega t}$ with wavevector ${{{\mathbf{k}}}}_{{{\mathrm{a}}}}$, a nanoantenna scatters some amount of power towards a point of interest **r**_0_. The time-independent total electric field phasor, ${{{\mathbf{E}}}}_{{{\mathrm{t}}}}({{{\mathbf{r}}}}_0)$, is the sum of the exciting and scattered fields and **r**_0_ will foster a time-independent cold spot if ${{{\mathbf{E}}}}_{{{\mathrm{t}}}}\left( {{{{\mathbf{r}}}}_0} \right) = 0$. Equating the three components of ${{{\mathbf{E}}}}_{{{\mathrm{t}}}}({{{\mathbf{r}}}}_0)$ to zero, we build a system of three independent equations, linear with respect to the variables offered by ${{{\mathbf{E}}}}_{{{\mathrm{a}}}}$. A cold spot, held under a non-zero exciting field, can be created anywhere **r**_0_ points to if there are at least four variables provided to the three scalar equations in ${{{\mathbf{E}}}}_{{{\mathrm{t}}}}\left( {{{{\mathbf{r}}}}_0} \right) = 0$. These three equations are what we refer to as the linear system of equations needing solved. By itself, the electric field vector ${{{\mathbf{E}}}}_{{{\mathrm{a}}}}$ enjoys only two degrees of freedom. The transversality condition restricts it to the plane perpendicular to ${{{\mathbf{k}}}}_{{{\mathrm{a}}}}$, so that it is always fully expressed as the sum of two components; a complex amplitude, $x_i$, multiplied by an in-plane basis vector, ${{{\hat{\mathbf e}}}}_i$, for $i = 1,2$ [Eq. 1],

1 $${{{\mathbf{E}}}}_{{{\mathrm{a}}}}\left( {{{\mathbf{r}}}} \right) = \left( {x_1{{{\hat{\mathbf e}}}}_1 + x_2{{{\hat{\mathbf e}}}}_2} \right){{{\mathrm{e}}}}^{i{{{\mathbf{k}}}}_{{{\mathrm{a}}}} \cdot {{{\mathbf{r}}}}}$$

One degree of freedom in this context translates to one variable in the system of equations ${{{\mathbf{E}}}}_{{{\mathrm{t}}}}\left( {{{{\mathbf{r}}}}_0} \right) = 0$; a single plane wave cannot buy us a non-trivial solution. If a second plane wave, ${{{\mathbf{E}}}}_{{{\mathrm{b}}}}\left( {{{\mathbf{r}}}} \right) = \left( {x_3{{{\hat{\mathbf e}}}}_3 + x_4{{{\hat{\mathbf e}}}}_4} \right){{{\mathrm{e}}}}^{i{{{\mathbf{k}}}}_{{{\mathrm{b}}}} \cdot {{{\mathbf{r}}}}}$, shines on the nanoantenna, then four variables in total are contributed to the linear system of equations, surpassing the number of conditions needing satisfied (three). Because of the linearity of Maxwell’s equations, the total field evaluated at ${{{\mathbf{r}}}}_0$ can be written as the linear sum of $x_i$ times the electric fields ${{{\mathbf{E}}}}_i({{{\mathbf{r}}}}_0)$ which $x_i$ are directly responsible for setting up (for instance, ${{{\mathbf{E}}}}_3({{{\mathbf{r}}}}_0)$ is the total field that exists at ${{{\mathbf{r}}}}_0$ when $x_3 = 1$ and all other plane wave components are zero) [Eq. 2],

2 $${{{\mathbf{E}}}}_{{{\mathrm{t}}}}({{{\mathbf{r}}}}_0) = x_1{{{\mathbf{E}}}}_1\left( {{{{\mathbf{r}}}}_0} \right) + x_2{{{\mathbf{E}}}}_2\left( {{{{\mathbf{r}}}}_0} \right) + x_3{{{\mathbf{E}}}}_3\left( {{{{\mathbf{r}}}}_0} \right) + x_4{{{\mathbf{E}}}}_4({{{\mathbf{r}}}}_0)$$

This equation may be compactly written in matrix form, when the vectors ${{{\mathbf{E}}}}_i({{{\mathbf{r}}}}_0)$ form the columns of the $3 \times 4$ coefficient matrix ${{{\mathbf{A}}}}\left( {{{{\mathbf{r}}}}_0} \right)$, and $x_i$ belong to the column vector ${{{\mathbf{x}}}}$ [Eq. 3],

3 $${{{\mathbf{A}}}}\left( {{{{\mathbf{r}}}}_0} \right){{{\mathbf{x}}}} = {{{\mathbf{E}}}}_{{{\mathrm{t}}}}({{{\mathbf{r}}}}_0)$$

It is possible to fix a cold spot at any ${{{\mathbf{r}}}}_0$ by calculating the null space of ${{{\mathbf{A}}}}\left( {{{{\mathbf{r}}}}_0} \right)$. In fact, any arrangement of particles of any shape and size, illuminated by two plane waves of any wavevector, has a $3 \times 4$ coefficient matrix of its own and a corresponding null space which creates a cold spot at ${{{\mathbf{r}}}}_0$. The variables $x_{1 - 4}$ are the four key ingredients needed to control the position of a single cold spot and can be sourced from any combination of incident fields; four TE polarised fields make an equally valid illuminating field, for example. More degrees of freedom can enforce more conditions, such as those which create simultaneous cold spots or control the derivatives of the total field at different points in space.

To summarise, one can enforce a zero-magnitude cold spot at any position ${{{\mathbf{r}}}}_0$ by first finding the individual electric fields, ${{{\mathbf{E}}}}_1({{{\mathbf{r}}}}_0)$, ${{{\mathbf{E}}}}_2({{{\mathbf{r}}}}_0)$, ${{{\mathbf{E}}}}_3({{{\mathbf{r}}}}_0)$ and ${{{\mathbf{E}}}}_4({{{\mathbf{r}}}}_0)$, developed by each of four degrees of freedom (the linear components of two incoming plane waves, for example). These fields may be found with any available method, be it analytical or numerical. Writing ${{{\mathbf{E}}}}_i({{{\mathbf{r}}}}_0)$ as the columns of a 3 × 4 matrix ${{{\mathbf{A}}}}\left( {{{{\mathbf{r}}}}_0} \right)$, the complex amplitude of each degree of freedom which produces the cold spot is given in the nullspace ${{{\mathbf{x}}}}$ of ${{{\mathbf{A}}}}\left( {{{{\mathbf{r}}}}_0} \right)$. So far, we have not imposed any restrictions on the geometry or material of the nanoantenna. The simple linear algebra concepts, which describe how two plane waves’ electric field vectors can design a zero at any location, function as long as [Eq. 2] holds true which, provided no non-linear materials, it does in any volume of space under illumination by two plane waves. No mention of how the plane waves are scattered (if at all) is needed. A resonant nanoantenna simply serves to rupture the interference pattern set up by the plane waves, sculpting its planar zero nodes into point-like, high contrast cold spots.

### Demonstrations

**Fig. 2:**
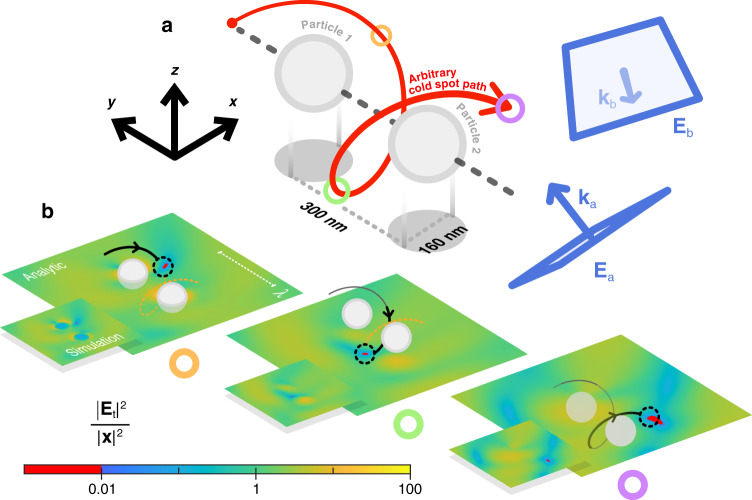
Moving a cold spot around two silver point scatterers in a theoretical framework **a** Helical path taken by a cold spot around two silver nanoparticles, illuminated by two plane waves, ${{{\mathbf{E}}}}_{{{\mathrm{a}}}}$ and ${{{\mathbf{E}}}}_{{{\mathrm{b}}}}$, with wavevectors ${{{\mathbf{k}}}}_{{{\mathrm{a}}}} = k\left( {\frac{{\sqrt 3 }}{2}{{{\hat{\mathbf y}}}} + \frac{1}{2}{{{\hat{\mathbf z}}}}} \right)$ and ${{{\mathbf{k}}}}_{{{\mathrm{b}}}} = k\left( {\frac{{\sqrt 2 }}{{\sqrt 7 }}{{{\hat{\mathbf x}}}} + \frac{1}{{\sqrt 7 }}{{{\hat{\mathbf y}}}} - \frac{2}{{\sqrt 7 }}{{{\hat{\mathbf z}}}}} \right)$. The free space wavenumber $k = \frac{{2\pi }}{\lambda }$ with $\lambda = 500$ nm. The cold spot is moved along the path by changing $x_{1 - 4}$, manifesting as the polarisation, relative intensity, and phase of ${{{\mathbf{E}}}}_{{{\mathrm{a}}}}$ and ${{{\mathbf{E}}}}_{{{\mathrm{b}}}}$. **b** Snapshots of the surrounding electric field obtained at three positions on the helical path, marked by the coloured rings in (a). In the measured quantity, $\frac{{\left| {{{{\mathbf{E}}}}_{{{\mathrm{t}}}}} \right|^2}}{{\left| {{{\mathbf{x}}}} \right|^2}}$, $\left| {{{{\mathbf{E}}}}_{{{\mathrm{t}}}}} \right|^{2}$ is the intensity of the total electric field and $\left| {{{\mathbf{x}}}} \right|^2\,=\,|{{{\mathbf{E}}}}_{{{\mathrm{a}}}}|^2\,+\,|{{{\mathbf{E}}}}_{{{\mathrm{b}}}}|^2$ is the squared magnitude of the solution to ${{{\mathbf{A}}}}\left( {{{{\mathbf{r}}}}_0} \right){{{\mathbf{x}}}} = 0$. The inset distributions present numerically simulated results of the same scenario, with plane wave polarisations identical to the analytical case. An animated version of this figure is found in the supplementary information

As long as we understand how each incident field polarisation component interacts with a nanoantenna and develops a total electric field throughout space, we can make a cold spot at any position **r**_0_. One might do this for a general nanoantenna with a complicated analytical model, like the discrete dipole approximation, or in a far less time-consuming manner using numerical simulations. Whichever method is chosen is unimportant; what matters is that the electric fields ${{{\mathbf{E}}}}_i({{{\mathbf{r}}}}_0)$ of [Eq. 2] associated with the nanoantenna and incident fields are found. We first present a basic analytical model, where a cold spot is moved in an arbitrarily chosen helical path around two coupled dipolar scatterers, under illumination by two plane waves ${{{\mathbf{E}}}}_{{{\mathrm{a}}}}$ and ${{{\mathbf{E}}}}_{{{\mathrm{b}}}}$. We avoid modelling a single point scatterer to prevent a situation where, at a certain distance from the dipole, the cold spot explodes into a zero plane because the dipole falls into a node of the incident interference pattern ${{{\mathbf{E}}}}_{{{\mathrm{a}}}}\left( {{{\mathbf{r}}}} \right) + {{{\mathbf{E}}}}_{{{\mathrm{b}}}}\left( {{{\mathbf{r}}}} \right)$. The two wavevectors ${{{\mathbf{k}}}}_{{{\mathrm{a}}}}$ and ${{{\mathbf{k}}}}_{{{\mathrm{b}}}}$, given in the caption of Fig. 2(a), are chosen arbitrarily, with one important consideration made to avoid mirror-symmetric scattering of ${{{\mathbf{E}}}}_{{{\mathrm{a}}}}$ and ${{{\mathbf{E}}}}_{{{\mathrm{b}}}}$. This is simply that ${{{\mathbf{k}}}}_{{{\mathrm{a}}}}$ and ${{{\mathbf{k}}}}_{{{\mathrm{b}}}}$ are not anti-parallel (${{{\mathbf{k}}}}_{{{\mathrm{a}}}} = - {{{\mathbf{k}}}}_{{{\mathrm{b}}}}$) and aligned with a symmetry axis of the two separated dipoles. If this were the case, then any attempt to force a cold spot at a certain location ${{{\mathbf{r}}}}_0$ would result in a pair of cold spots, one at ${{{\mathbf{r}}}}_0$, and the other in a symmetric position. Our otherwise arbitrary choice of ${{{\mathbf{k}}}}_{{{\mathrm{a}}}}$ and ${{{\mathbf{k}}}}_{{{\mathrm{b}}}}$ develop an asymmetric electric field around the dipoles and ensure the uniqueness of the exact zero. Further mathematical details, including use of the coupled dipole model to derive the columns ${{{\mathbf{E}}}}_i({{{\mathbf{r}}}}_0)$ of the system matrix ${{{\mathbf{A}}}}\left( {{{{\mathbf{r}}}}_0} \right)$ for any number of plane waves incident on any number of dipolar scatterers, are found in the supplementary information. A far more complicated analytical model of any nanoantenna geometry could be built using the discrete dipole approximation.

With both dipoles suspended in free space and separated by 300 nm, the wavelength of the plane waves was $\lambda = 500$ nm. The Mie polarisability of the dipoles was calculated as though they were identical silver nanospheres ($\varepsilon _{{{{\mathrm{Ag}}}}} = - 8.28 + 0.78i$ at the incident wavelength^36^) of 80 nm radius. The arrangement is shown in Fig. 2(a). By tuning only the polarisation, relative intensity and phase of the plane waves ${{{\mathbf{E}}}}_{{{\mathrm{a}}}}$ and ${{{\mathbf{E}}}}_{{{\mathrm{b}}}}$, via the complex variables $x_{1 - 4}$, a cold spot was moved along a helical path. The total electric field ${{{\mathbf{E}}}}_{{{\mathrm{t}}}}\left( {{{\mathbf{r}}}} \right)$, was found by adding the exact field radiated from two coupled electric dipoles to the combined incident field, ${{{\mathbf{E}}}}_{{{\mathrm{a}}}}\left( {{{\mathbf{r}}}} \right) + {{{\mathbf{E}}}}_{{{\mathrm{b}}}}\left( {{{\mathbf{r}}}} \right)$. In three different stages in its journey, plots of the intensity enhancement on the *xy* plane coinciding with the cold spot are shown in Fig. 2(b). The intensity enhancement describes how many times stronger the local power density, $\left| {{{{\mathbf{E}}}}_{{{\mathrm{t}}}}} \right|^2$, is than that of the combined incident field $\left| {{{{\mathbf{E}}}}_{{{\mathrm{a}}}}} \right|^2 + \left| {{{{\mathbf{E}}}}_{{{\mathrm{b}}}}} \right|^2$, which is the magnitude squared of the solution to [Eq. 3], $\left| {{{\mathbf{x}}}} \right|^2$. Given in a logarithmic scale, the plotted quantity is proportional to the logarithmic electric field magnitude; the colour map reveals the regions of high and low intensity set up by the sum of the incident and dipolar fields. Our analytical calculations are verified by numerical simulations in *CST Microwave Studio*, the results given in the inset of each field distribution. For each of the three presented cold spot positions, a simulation of the two-dipole, two-plane wave arrangement, including the analytically calculated polarisation states of the plane waves, was performed with physical silver nanospheres in place of the dipoles. These simulations check the correctness of our analytical calculations, and the validity of the dipole approximation they use, by replicating the analytical model perfectly save for the exact spherical geometry of the particles, which support (albeit small) higher order multipoles and have a realistic internal field. The cold spot is seen clearly in each inset; our analytical and numerical results are in excellent agreement.

**Fig. 3:**
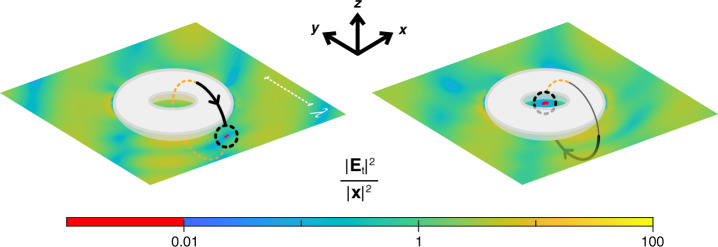
Moving a cold spot around a silver torus in simulations Results obtained for a torus particle, via numerical simulations. The intensity enhancement on the *xy* plane containing a cold spot, moved along a circular path (angled into the page) by the polarisation, relative intensity and phase of two plane waves with $\lambda = 800$ nm, is plotted at two stages in the cold spot’s journey. The complete cold spot trajectory is animated in the supplementary material

In a second scenario, two plane waves are incident in free space on a silver toroidal particle with the same wavevector directions as ${{{\mathbf{k}}}}_{{{\mathrm{a}}}}$ and ${{{\mathbf{k}}}}_{{{\mathrm{b}}}}$ of the analytical model, only this time with an 800 nm wavelength. A cold spot is created and sent along a circular trajectory, threading the torus. Despite the significantly more complicated particle geometry, numerical simulations make this no more difficult to achieve than a moving cold spot in our simple analytical model; the specifics of the particle’s interaction with incident light makes no difference. We take a numerical approach to forcing a cold spot by finding the electric fields ${{{\mathbf{E}}}}_i({{{\mathbf{r}}}}_0)$ of [Eq. 2], and therefore the coefficient matrix ${{{\mathbf{A}}}}\left( {{{{\mathbf{r}}}}_0} \right)$, using simulations rather than an analytical calculation. In the nullspace **x** of ${{{\mathbf{A}}}}\left( {{{{\mathbf{r}}}}_0} \right)$, we find the plane wave polarisation components which develop a cold spot at ${{{\mathbf{r}}}}_0$. We then simulate this specific polarisation excitation with the two plane waves to verify the cold spot’s successful creation. The cold spot is moved along the circular path in Fig. 3 by changing ${{{\mathbf{r}}}}_0$ in steps, re-solving for **x**, and re-simulating the torus with each new plane wave polarisation.

These results are a reflection of the generality of [Eq. 2]; that an electric field zero can be enforced around any linear light-scattering matter using simple linear algebra techniques, hinging on four degrees of freedom supplied by an arrangement of incident electric fields. An important next step is to try to define the size and shape of the moving cold spot.

### Cold spot characterization

**Fig. 4:**
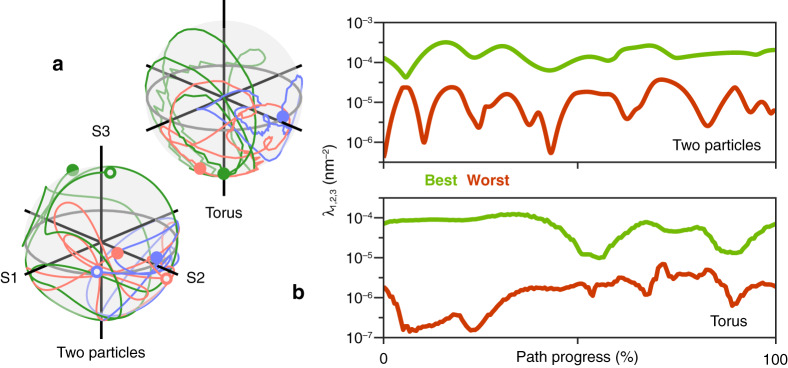
Characterisation of incident fields and cold spots **a** Poincaré spheres showing the changes in each plane wave’s polarisation (red line: ${{{\mathbf{E}}}}_{{{\mathrm{a}}}}$, blue line: ${{{\mathbf{E}}}}_{{{\mathrm{b}}}}$) as the cold spot is moved along the two-particle and torus paths. The hollow and filled circles represent the start and end polarisations for the cold spot path (these are the same for the torus). The green line gives the relative phase and amplitude between the plane waves on a superimposed Bloch sphere. The axes are ${{{\mathrm{S}}}}1 = \left| {x_m} \right|^2 - \left| {x_n} \right|^2$, ${{{\mathrm{S}}}}2 = 2{{{\mathrm{Re}}}}\left\{ {x_mx_n^ \ast } \right\}$, and ${{{\mathrm{S}}}}3 = - 2{{{\mathrm{Im}}}}\left\{ {x_mx_n^ \ast } \right\}$, where for the red, blue, and green lines, $x_m,x_n = x_1,x_2$, $x_m,x_n = x_3,x_4$ and $x_m,x_n = x_1,x_3$ respectively. **b** Biggest (best-case) and smallest (worst-case) of the eigenvalues ($\lambda _{1,2,3}$) of **B**, the matrix which characterises the cold spot shape, over the course of the two-particle and torus paths. Expanding the electric field in the cold spot and calculating the nearby field intensity, the cold spot is seen to be ellipsoidal. The eigenvalues of **B** represent the reciprocals of the squares of the semi-axes of the ellipsoid enclosing the cold spot, the ellipsoid surface defined at $\frac{{\left| {{{{\tilde{\mathbf E}}}}_{{{\mathrm{t}}}}\left( {{{\mathbf{r}}}} \right)} \right|^2}}{{\left| {{{\mathbf{x}}}} \right|^2}} = 1$

In Fig. 4, we characterise the incident plane waves and cold spots of Fig. 2 and Fig. 3. In Fig. 4(a), the changing polarisations of the two plane waves (red and blue lines) responsible for moving the cold spot are mapped to the Poincaré sphere for the circular (torus) and helical (two-particle) paths. The green line encodes the relative amplitude and phase offset between the plane waves on a superimposed Bloch sphere. Meanwhile, a measure of the size of the cold spots of the helical and circular paths is given by Fig. 4(b). While a hot spot’s size can be measured by its full width at half maximum, it is not easy to appreciate the size of a cold spot with an exact zero at its centre. We might begin by approximating the field nearby with a first order Taylor expansion, ${{{\tilde{\mathbf E}}}}_{{{\mathrm{t}}}}\left( {{{\mathbf{r}}}} \right) = {{{\mathbf{Jv}}}}$, where **J** is the Jacobian of ${{{\mathbf{E}}}}_{{{\mathrm{t}}}}\left( {{{\mathbf{r}}}} \right)$ evaluated at the cold spot centre, ${{{\mathbf{r}}}}_0$, and ${{{\mathbf{v}}}} = {{{\mathbf{r}}}} - {{{\mathbf{r}}}}_0$. Computing $\frac{{\left| {{{{\tilde{\mathbf E}}}}_{{{\mathrm{t}}}}\left( {{{\mathbf{r}}}} \right)} \right|^2}}{{\left| {{{\mathbf{x}}}} \right|^2}}$ and equating to a constant value $\frac{{\left| {{{{\tilde{\mathbf E}}}}_{{{\mathrm{t}}}}} \right|^2}}{{\left| {{{\mathbf{x}}}} \right|^2}}$ returns the equation of an ellipsoid which encloses the cold spot [Eq. 4],

4 $${{{\mathbf{v}}}}^T{{{\mathbf{Bv}}}} = \frac{{\left| {{{{\tilde{\mathbf E}}}}_{{{\mathrm{t}}}}} \right|^2}}{{\left| {{{\mathbf{x}}}} \right|^2}}$$

The cold spot is characterised by the real, positive-definite matrix ${{{\mathbf{B}}}} = \frac{1}{{\left| {{{\mathbf{x}}}} \right|^2}}{{{\mathrm{Re}}}}\left\{ {{{{\mathbf{J}}}}^T{{{\mathbf{J}}}}^ \ast } \right\}$; its eigenvectors reveal the orientation of the ellipsoid’s principle axes and its eigenvalues are the quadratic coefficients in the equation $\lambda _1\left( {x^\prime - x_0^\prime } \right)^2 + \lambda _2\left( {y^\prime - y_0^\prime } \right)^2 + \lambda _3\left( {z^\prime - z_0^\prime } \right)^2 = \frac{{\left| {{{{\tilde{\mathbf E}}}}_{{{\mathrm{t}}}}\left( {{{\mathbf{r}}}} \right)} \right|^2}}{{\left| {{{\mathbf{x}}}} \right|^2}}$, where $x\prime$, $y\prime$, and $z\prime$ are aligned to the ellipsoid semi-axes. The intensity enhancement $\frac{{\left| {{{{\tilde{\mathbf E}}}}_{{{\mathrm{t}}}}\left( {{{\mathbf{r}}}} \right)} \right|^2}}{{\left| {{{\mathbf{x}}}} \right|^2}}$ increases in quadratic fashion along $x\prime$, $y\prime$, and $z\prime$, from zero in the cold spot at ${{{\mathbf{r}}}}_0^\prime$. The larger the eigenvalue, measured in nm^−2^, the more confined the cold spot is in the direction of the corresponding eigenvector. The biggest (best-case) and smallest (worst-case) eigenvalues belonging to the cold spot ellipsoid are plotted in Fig. 4(b) as the cold spot moves along the two-particle and torus paths. In the best-case direction, our three-dimensional cold spot in either path is comparable to or smaller than a narrow, two-dimensional doughnut beam zero used for fluorescent imaging^37^, while the worst-case eigenvalue is in general between one and two orders of magnitude smaller. The cold spot might be tightened significantly by optimising the particle-plane wave arrangement. Improving particle coupling by adjusting separation, the strength of the scattered fields by tuning the particle geometry and ${{{\mathbf{k}}}}_{{{\mathrm{a}}}}$ and ${{{\mathbf{k}}}}_{{{\mathrm{b}}}}$, or simply increasing the number of particles in the system could produce smaller cold spots with a more consistent ellipsoidal shape.

### Sensitivity to perturbation

We have so far relied on prior knowledge of the nanoantenna geometry, position, and permittivity, used either in an analytical calculation or numerical simulation, to produce a cold spot somewhere in its near field. Under the solved-for plane wave amplitudes $x_i$, the cold spot is disturbed if a nanoantenna parameter deviates slightly, evaporating completely if the nanoantenna wanders too far from its supposed position or is the wrong material. This raises the interesting possibility that with a suitable probe, our technique becomes a mechanism for nanoparticle position and material sensing.

**Fig. 5:**
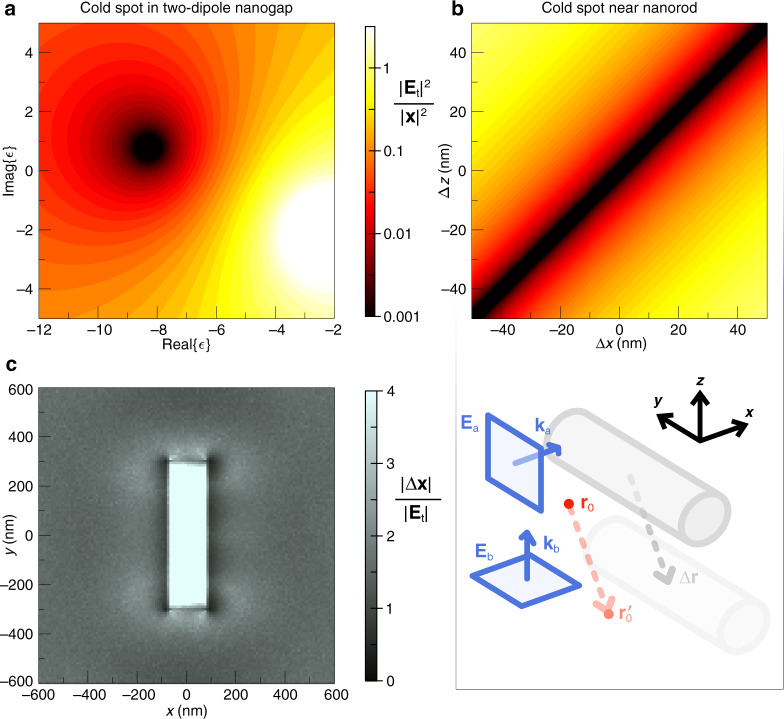
Perturbation response **a** Response of a cold spot, positioned exactly between the two dipolar scatterers treated previously, to changes in the real and imaginary parts of the scatterers’ permittivity. The dipoles are under constant illumination by two plane waves with amplitudes which assume $\varepsilon _{{{{\mathrm{Ag}}}}} = - 8.28 + 0.78i$. **b** Response of a cold spot in its relative position ${{{\mathbf{r}}}}_0 + \Delta {{{\mathbf{r}}}}$, where ${{{\mathbf{r}}}}_0 = - 200{{{\hat{\mathbf x}}}}$ nm, to a silver nanorod oriented along ${{{\hat{\mathbf y}}}}$ and originally centred at the origin, when the nanorod is displaced by a vector $\Delta {{{\mathbf{r}}}} = \Delta x{{{\hat{\mathbf x}}}} + \Delta z{{{\hat{\mathbf z}}}}$. **c** The total error in the illuminating plane waves, $\left| {\Delta {{{\mathbf{x}}}}} \right|$, which causes a unit rise in the electric field magnitude in the cold spot, $\left| {{{{\mathbf{E}}}}_{{{\mathrm{t}}}}} \right|$, calculated for every cold spot position in the *z* = 0 cross section of the silver nanorod of (**b**). Brighter pixels highlight positions where the cold spot is less sensitive to plane wave error, while darker pixels represent more error-sensitive locations

To test the response of a cold spot to discrepancy in scatterer permittivity, a fixed cold spot was designed for an ${{{\mathbf{r}}}}_0$ in middle of the nanogap of the two silver point scatterers of Fig. 2(a), the incident wavelength once again 500 nm (a cold spot in *ε* space at $\varepsilon _{{{{\mathrm{Ag}}}}} = - 8.28 + 0.78i$). Having obtained *x*_*i*_, the plane waves’ cold spot-making electric field vectors were kept constant while the real and imaginary parts of the particle permittivity were changed. This way, the total electric field, ${{{\mathbf{E}}}}_{{{\mathrm{t}}}}\left( {{{{\mathbf{r}}}}_0,\varepsilon } \right)$, was probed throughout *ε* space at the fixed real space position ${{{\mathbf{r}}}}_0$, and the intensity enhancement plotted in Fig. 5(a). A reasonably dark cold spot $\left( {\frac{{\left| {{{{\mathbf{E}}}}_{{{\mathrm{t}}}}} \right|^2}}{{\left| {{{\mathbf{x}}}} \right|^2}} < 0.01} \right)$ is shown to exist within about ±1 of the real and imaginary parts of $\varepsilon _{{{{\mathrm{Ag}}}}}$. If the two point scatterers are a different material, such as gold with $\varepsilon _{{{{\mathrm{Au}}}}} = - 2.77 + 3.18i$ at $\lambda = 500$ nm^36^, the cold spot vanishes. This cold spot permittivity sensitivity also applies to the background. The cold spot fades if the surrounding effective refractive index deviates from what is expected, as might occur in the presence of organic particles (like viruses) and in other biological and chemical sensing scenarios. A brief test of this cold spot behaviour, demonstrating sensitivity to subtle changes in background refractive index, is provided in the supplemental material.

We next assess the response of a cold spot to a displaced nanoantenna. We consider a silver nanorod of 600 nm length in free space, oriented along ${{{\hat{\mathbf y}}}}$, centred at the origin and with 160 nm diameter, under illumination by two, $\lambda = 500$ nm plane waves in *CST Microwave Studio* simulations. The plane waves have wavevectors ${{{\mathbf{k}}}}_{{{\mathrm{a}}}} = k{{{\hat{\mathbf x}}}}$ and ${{{\mathbf{k}}}}_{{{\mathrm{b}}}} = k{{{\hat{\mathbf z}}}}$ and enforce a cold spot at ${{{\mathbf{r}}}}_0 = - 200{{{\hat{\mathbf x}}}}$ nm, as shown in the lower diagram of Fig. 5(b). If we displace the nanorod by a vector $\Delta {{{\mathbf{r}}}}$, and track the electric field in a position fixed relative to the nanoantenna, ${{{\mathbf{r}}}}_0^\prime = {{{\mathbf{r}}}}_0 + \Delta {{{\mathbf{r}}}}$, then a cold spot at ${{{\mathbf{r}}}}_0^\prime$ is robust to displacements $\Delta {{{\mathbf{r}}}}\parallel {{{\mathbf{k}}}}_{{{\mathrm{a}}}} \times {{{\mathbf{k}}}}_{{{\mathrm{b}}}}$ and $\Delta {{{\mathbf{r}}}}\parallel {{{\mathbf{k}}}}_{{{\mathrm{a}}}} + {{{\mathbf{k}}}}_{{{\mathrm{b}}}}$ because along these vectors, the two plane waves do not advance in phase at all, or they advance in phase by the same amount. The intensity enhancement at ${{{\mathbf{r}}}}_0^\prime$ is calculated in the upper plot of Fig. 5(b) for nanorod displacements $\Delta {{{\mathbf{r}}}} = \Delta x{{{\hat{\mathbf x}}}} + \Delta z{{{\hat{\mathbf z}}}}$ in the *xz* plane, which contains both incident wavevectors. The obvious feature of Fig. 5(b) is the dark stripe which stretches over $\Delta {{{\mathbf{r}}}}$ parallel to ${{{\hat{\mathbf x}}}} + {{{\hat{\mathbf z}}}}$; under these displacements, the nanorod tows the cold spot away from its original absolute position, whereas the cold spot is sensitive to displacements $\Delta {{{\mathbf{r}}}} \bot {{{\hat{\mathbf x}}}} + {{{\hat{\mathbf z}}}}$.

Finally, we disturb the cold spot via perturbations in the incident plane waves. If there is a random complex error $\Delta {{{\mathbf{x}}}}$ added to the calculated complex amplitude of each plane wave component, held in the solution **x**, then the cold spot becomes blurred and a non-zero electric field is left behind at ${{{\mathbf{r}}}}_0$. From (3), this residual electric field is equal to ${{{\mathbf{A}}}}\left( {{{{\mathbf{r}}}}_0} \right)\left( {{{{\mathbf{x}}}} + \Delta {{{\mathbf{x}}}}} \right) = {{{\mathbf{A}}}}\left( {{{{\mathbf{r}}}}_0} \right)\Delta {{{\mathbf{x}}}}$ (since **x** is the nullspace of ${{{\mathbf{A}}}}\left( {{{{\mathbf{r}}}}_0} \right)$), and depends therefore on the size of the error added to the elements of **x** and the position of the cold spot, ${{{\mathbf{r}}}}_0$. To visualise a cold spot’s position-dependent response to plane wave error, a map of the *xy* plane slicing through the previous silver nanorod is given in Fig. 5(c). After creating a cold spot at each pixel, 100 samples of random error are added to the solved-for plane wave components, and the residual field magnitude left in the cold spot is averaged for a range of different error magnitudes, $\left| {\Delta {{{\mathbf{x}}}}} \right|$. The colour of each pixel corresponds to $\frac{{\left| {\Delta {{{\mathbf{x}}}}} \right|}}{{\left| {{{{\mathbf{E}}}}_{{{\mathrm{t}}}}} \right|}}$, which is the error magnitude in the two plane waves which results in a unit increase in the electric field magnitude in the cold spot. If a cold spot is to be made in a position in Fig. 5(c) with $\frac{{\left| {\Delta {{{\mathbf{x}}}}} \right|}}{{\left| {{{{\mathbf{E}}}}_{{{\mathrm{t}}}}} \right|}} = 3$, for instance, then a total plane wave error of 3 arb. u. will produce a non-zero cold spot with a 1 arb. u. magnitude electric field. In the position of the brighter pixels, the cold spot is more tolerant of plane wave error, while the darker pixels represent much more error-sensitive positions. Since the fields ${{{\mathbf{E}}}}_i({{{\mathbf{r}}}}_0)$ of [Eq. 2] can be found for **r**_0_ inside the nanorod, we can create cold spots there too. A nanorod of this size’s inner electric field is naturally low due to losses, meaning internal cold spots are much less sensitive to plane wave error (hence the bright solid bar of the nanorod cross section). Meanwhile, high-contrast cold spots created on top of the nanorod’s native hot spots (at the corners; the outer circumference of each end of the nanorod) are very sensitive to plane wave error.

### Cold spot pulse-shaping

**Fig. 6:**
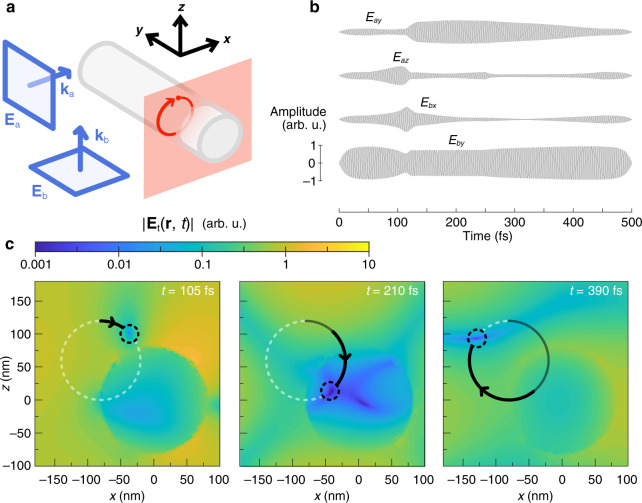
Pulsed cold spot (a) Diagram of the silver nanorod and the circular path in the *γ* = −200 nm plane, along which a cold spot is moved in the time domain by four modulated pulses. (b) Pulse shapes of the ${{{\hat{\mathbf y}}}}$ and ${{{\hat{\mathbf z}}}}$ components of ${{{\mathbf{E}}}}_{{{\mathrm{a}}}}$ (top two waves) and the ${{{\hat{\mathbf x}}}}$ and ${{{\hat{\mathbf y}}}}$ components of ${{{\mathbf{E}}}}_{{{\mathrm{b}}}}$ (bottom two waves) responsible for the movement of the cold spot. A reference amplitude scale to which each pulse is drawn is given next to the bottom pulse in arbitrary units. (c) Three snapshots of the time-dependent total electric field magnitude, calculated in a time-domain simulation in *CST Microwave Studio*, on a section of the $y = - 200$ nm plane in arbitrary units. Each snapshot is taken at the indicated time and shows the cold spot at a different stage along the circular path. A complete video of the moving cold spot is given in the supplemental material

Cold spots can also be manipulated under non time-harmonic incident signals. For a truly ultra-fast command over the nanoantenna near field, we can encode the entire path of a moving cold spot into four modulated pulses, one per degree of freedom. We must stress that in this section, we simply raise the possibility of ultra-fast, time-domain cold spot control, and do not discuss the practicality of generating the pulses with existing technology. This cannot be guaranteed, though the science and techniques of ultra-fast pulse shaping is a fast-growing field. A time-harmonic approximation, through which we arrive at [Eq. 3], gives a reasonable description of a time-domain pulse only if its amplitude and phase vary slowly over time compared to the designed incident frequency. This has consequences for the clarity of the cold spot. Here we test the limits of the time-harmonic approximation, showing that a clear cold spot can be moved even by short pulses. We enforce the time-dependent position of a cold spot, ${{{\mathbf{r}}}}_0\left( t \right)$, with two pairs of pulses, designed under approximation throughout space as $x_{1,2}{{{\mathrm{e}}}}^{i{{{\mathbf{k}}}}_{{{\mathrm{a}}}} \cdot {{{\mathbf{r}}}}}{{{\hat{\mathbf e}}}}_{1,2}$ and $x_{3,4}{{{\mathrm{e}}}}^{i{{{\mathbf{k}}}}_{{{\mathrm{b}}}} \cdot {{{\mathbf{r}}}}}{{{\hat{\mathbf e}}}}_{3,4}$ (although our simulation results account for propagation delay), each pair describing the time-varying orthogonal components of one plane wave. Note that it is not essential to use two plane waves here; one could instead pulse the nanoantenna with four linearly polarised plane waves. In Fig. 6, we present three snapshots of the electric field intensity around a 600 nm long, 160 nm diameter silver nanorod, oriented along ${{{\hat{\mathbf y}}}}$ and centred at the origin, as a cold spot was moved along the circular trajectory shown in Fig. 6(a). The cold spot path lies entirely in the *y* = −200 nm plane, and even passes through the nanorod. The polarisation and phase of two incident plane waves, both of wavelength *λ* = 500 nm and with wavevectors ${{{\mathbf{k}}}}_{{{\mathrm{a}}}} = k{{{\hat{\mathbf x}}}}$ and ${{{\mathbf{k}}}}_{{{\mathrm{b}}}} = k{{{\hat{\mathbf z}}}}$, were modulated according to the waveforms shown in Fig. 6(b). The top two pulse shapes correspond to the amplitudes in arbitrary units of plane wave a’s ${{{\hat{\mathbf y}}}}$ and ${{{\hat{\mathbf z}}}}$ components, and the bottom two the ${{{\hat{\mathbf x}}}}$ and ${{{\hat{\mathbf y}}}}$ component amplitudes of plane wave b (further details on generating the pulses are found in Materials and Methods). In Fig. 6(c), the time-dependent electric field magnitude is plotted on the plane containing the cold spot path, also in arbitrary units. A complete video of moving the cold spot is provided in the supplemental material. The pulses lasted for 300 carrier wave periods, about 500 fs, though the modulation could be squeezed into or stretched over a different pulse duration, this way tuning the balance between the travelling speed and darkness of the cold spot.

## Discussion

The polarisation, relative intensity, and phase of two plane waves with fixed direction yield enough degrees of freedom to make a zero-magnitude, time-independent cold spot anywhere in the space around a nanoantenna of any arbitrary shape. Our work proposes the technique to realise this control with three examples; in each, a linear system of equations is constructed and solved to find the plane wave components corresponding to a set of co-ordinates for the cold spot. In two of these examples, a cold spot is held under time harmonic fields and moved by changing the complex component amplitudes of two plane waves of constant frequency. In the third example, a moving cold spot’s path is encoded into four pulse shapes, two per plane wave, which together send the cold spot along a circular trajectory near a silver nanorod. We would like to emphasise this paper’s purpose of presenting the technique for complete cold spot control rather than the examples themselves; by no means are we limited to moving a cold spot in the given paths around a dimer, a toroidal particle, or a nanorod. Supplying more degrees of freedom to the system, extra conditions may be put in place in addition to those supporting the cold spot – controlling the derivatives of the electric field at a point, for example. Degrees of freedom may be earned from the electric field components of independent plane waves, or any other parameter like the incidence direction or scatterers’ relative position or geometry, when allowed to change. A manufactured cold spot is sensitive to small changes in a nanoantenna’s material or position, which can disturb or completely destroy it. With a suitable probe, this delicate cold spot characteristic could act as a nanoparticle permittivity and position sensor. We have shown a way to unlock complete control of the electric field around a structure, to create unique, three-dimensional, highly sub-wavelength and zero-magnitude cold spots anywhere in its surroundings, with simple and potentially ultra-fast modulation of the incoming fields’ polarisation and intensity.

## Materials and Methods

Our study adopts two kinds of calculations to create cold spots and verify their existence. The first is an analytical calculation, used in *MATLAB* to enforce cold spots in the space of a simple, two-dipole, two-plane wave model. The second involves numerical simulations in *CST Microwave Studio*, a commercial electromagnetic solver, of more complicated, exact particle geometries. Simulations may be conducted for time-harmonic and pulsed plane wave illumination. The time-domain solver, which uses the Finite Integration Technique (FIT) and a hexahedral mesh, was used to calculate results in both time-harmonic and pulsed illumination cases.

### Analytical results

The results which show a cold spot moved in a helical path around two point scatterers (Fig. 2(b)) were produced using a *MATLAB* code. We modelled the electric field scattered by the particles as the sum of two exact dipolar fields, ${{{\mathbf{E}}}}_{{{{\mathrm{sca}}}}}\left( {{{\mathbf{r}}}} \right) = \frac{{k^2}}{{\varepsilon _0}}{{{\mathbf{G}}}}\left( {{{{\mathbf{r}}}},{{{\mathbf{r}}}}_1} \right){{{\mathbf{p}}}}_1 + \frac{{k^2}}{{\varepsilon _0}}{{{\mathbf{G}}}}\left( {{{{\mathbf{r}}}},{{{\mathbf{r}}}}_2} \right){{{\mathbf{p}}}}_2$, where ${{{\mathbf{G}}}}\left( {{{{\mathbf{r}}}},{{{\mathbf{r}}}}_p} \right)$ is the dyadic Green’s function originating at the dipole location, ${{{\mathbf{r}}}}_p$, and the electric dipole moment ${{{\mathbf{p}}}}_p$ incorporated both the influence of incident fields and coupling effects. Choosing a cold spot position ${{{\mathbf{r}}}}_0$ on the path, the complex component amplitudes of the two incident plane wave phasors ${{{\mathbf{E}}}}_{{{\mathrm{a}}}}({{{\mathbf{r}}}})$ and ${{{\mathbf{E}}}}_{{{\mathrm{b}}}}({{{\mathbf{r}}}})$, which enforce the cold spot at ${{{\mathbf{r}}}}_0$, were found from the nullspace of ${{{\mathbf{A}}}}\left( {{{{\mathbf{r}}}}_0} \right)$. Illuminating with these designed plane waves, the total electric field was evaluated throughout space as the sum of the incident and scattered fields, ${{{\mathbf{E}}}}_{{{\mathrm{t}}}}\left( {{{\mathbf{r}}}} \right) = {{{\mathbf{E}}}}_{{{\mathrm{a}}}}\left( {{{\mathbf{r}}}} \right) + {{{\mathbf{E}}}}_{{{\mathrm{b}}}}\left( {{{\mathbf{r}}}} \right) + {{{\mathbf{E}}}}_{{{{\mathrm{sca}}}}}({{{\mathbf{r}}}})$, where ${{{\mathbf{E}}}}_{{{\mathrm{a}}}}\left( {{{\mathbf{r}}}} \right) = \left( {x_1{{{\hat{\mathbf e}}}}_1 + x_2{{{\hat{\mathbf e}}}}_2} \right){{{\mathrm{e}}}}^{i{{{\mathbf{k}}}}_{{{\mathrm{a}}}} \cdot {{{\mathbf{r}}}}}$ and ${{{\mathbf{E}}}}_{{{\mathrm{b}}}}\left( {{{\mathbf{r}}}} \right) = \left( {x_3{{{\hat{\mathbf e}}}}_3 + x_4{{{\hat{\mathbf e}}}}_4} \right){{{\mathrm{e}}}}^{i{{{\mathbf{k}}}}_{{{\mathrm{b}}}} \cdot {{{\mathbf{r}}}}}$. The coefficient matrix ${{{\mathbf{A}}}}\left( {{{{\mathbf{r}}}}_0} \right)$ may be found for a certain **r**_0_ using the expression for ${{{\mathbf{E}}}}_{{{\mathrm{t}}}}\left( {{{\mathbf{r}}}} \right)$; details are provided in the supplemental material. By advancing **r**_0_ in steps and repeating the solving process, the cold spot was moved along the helical path. Higher order multipoles in the particles were neglected; in either particle, the electric dipole coefficient was found to be the only non-negligible Mie scattering coefficient. To create the permittivity sensitivity plot Fig. 5(a), the total electric field was instead evaluated throughout *ε* space at a fixed real space position, exactly between the two dipoles.

### Numerical simulations (time-harmonic)

Time-harmonic simulations for the two particle, torus and nanorod scenarios were conducted in *CST Microwave Studio*. In the inset plots of Fig. 2(b), two physical silver nanospheres of 80 nm radius were simulated under illumination by the same two plane waves with the same polarisation as was calculated using the two-dipole analytical model. The torus and nanorod results were produced entirely numerically; both the nullspace **x** of ${{{\mathbf{A}}}}\left( {{{{\mathbf{r}}}}_0} \right)$, and the total field set up around the particles when under excitation by **x**, were found in simulations. To move a cold spot requires that the polarisation and relative amplitude and phase of both plane waves change. At first glance, this suggests that a very large number of individual simulations need performing to show a cold spot smoothly traversing space. By exploiting the linearity of Maxwell’s equations, however, the total number of simulations needed to create and reveal a cold spot in any position can be minimised to four. Only the electric fields ${{{\mathbf{E}}}}_1({{{\mathbf{r}}}})$, ${{{\mathbf{E}}}}_2({{{\mathbf{r}}}})$, ${{{\mathbf{E}}}}_3({{{\mathbf{r}}}})$, and ${{{\mathbf{E}}}}_4({{{\mathbf{r}}}})$ from [Eq. 2] need to be evaluated throughout space. The linear superposition of these electric fields, each multiplied by the corresponding incident complex component amplitude, can obtain the total electric field from any combination of plane wave amplitude, phase and polarisation states. Using this shortcut is exactly equivalent to conducting one individual simulation per cold spot position **r**_0_, wherein both plane waves are incident at the same time and polarised specifically to create a cold spot at **r**_0_.

### Numerical simulations (time-domain)

Time domain simulations were also performed in *CST Microwave Studio* to obtain the results of Fig. 6. Two plane waves with wavevectors ${{{\mathbf{k}}}}_{{{\mathrm{a}}}} = k{{{\hat{\mathbf x}}}}$ and ${{{\mathbf{k}}}}_{{{\mathrm{b}}}} = k{{{\hat{\mathbf z}}}}$ were incident on a 600 nm long, 160 nm diameter silver nanorod suspended in free space, its length parallel to ${{{\hat{\mathbf y}}}}$. The pulse shapes shown in Fig. 6(b) correspond to the components of each plane wave’s time-dependent electric field vector; the top two waveforms belong to ${{{\mathbf{E}}}}_{{{\mathrm{a}}}} = E_{ay}\left( t \right){{{\hat{\mathbf y}}}} + E_{az}\left( t \right){{{\hat{\mathbf z}}}}$, and the bottom two ${{{\mathbf{E}}}}_{{{\mathrm{b}}}} = E_{bx}\left( t \right){{{\hat{\mathbf x}}}} + E_{by}\left( t \right){{{\hat{\mathbf y}}}}$. These pulse shapes were used as the simulations’ excitation signals, and were first generated using a *MATLAB* code. Above, the time-harmonic simulations paragraph tells that the cold spot solution **x** may be found, using numerical simulations, for any position **r**_0_. Naturally, we can also find the position-dependent solution, ${{{\mathbf{x}}}}\left( {{{{\mathbf{r}}}}_0} \right)$, for all **r**_0_ in the $- 200{{{\hat{\mathbf y}}}}$ nm plane. Because simulation results have a finite resolution, we then interpolate ${{{\mathbf{x}}}}\left( {{{{\mathbf{r}}}}_0} \right)$ over the $- 200{{{\hat{\mathbf y}}}}$ nm plane, smoothing it between mesh cells. For a cold spot to move over time in the clockwise circular path *C* of Fig. 6(a), ${{{\mathbf{x}}}}\left( {{{{\mathbf{r}}}}_0} \right)$ for ${{{\mathbf{r}}}}_0 \in C$ can be extracted and made a function of time, ${{{\mathbf{x}}}}_C(t)$. The speed that the cold spot completes the path, relative to the incident plane wave period, may be adjusted in ${{{\mathbf{x}}}}_C(t)$ as desired (possibly with further interpolation). Finally, the four pulse shapes are the rows of ${{{\mathrm{Re}}}}\left\{ {{{{\mathbf{x}}}}_C(t){{{\mathrm{e}}}}^{ - i\omega t}} \right\}$, where $\omega = \frac{{2\pi }}{\lambda }$, and *λ* = 500 nm.

## Supplementary information

Creating and Moving Nanoantenna Cold Spots Anywhere: Supplementary Information

Moving Cold Spot: Two Dipoles

Moving Cold Spot: Torus Particle

Cold spot animation: time-domain
