# Supplementary material for: Creating and moving nanoantenna cold spots anywhere
Source: Light Sci Appl. 2022 Aug 30;11:258. doi: 10.1038/s41377-022-00893-7 (PMC9427961; doi:10.1038/s41377-022-00893-7)
Supplement: Supplementary file 1 — Creating and Moving Nanoantenna Cold Spots Anywhere: Supplementary Information [file 41377_2022_893_MOESM1_ESM.pdf]

Creating and Moving Nanoantenna Cold Spots Anywhere: Supplementary Information

Alex J. Vernon   Francisco J. Rodríguez-Fortuño

Department of Physics, King's College London, Strand, London WC2R 2LS, United Kingdom

1 Constructing $\mathbf{A}(\mathbf{r}_0)$

A plane wave carries two degrees of freedom into the set of equations which describe the electric field developed around a nanoantenna. The linearity of Maxwell's equations means that this electric field can be expressed as a linear combination of the fields set up directly by plane wave's individual components. With N incident plane waves, the total electric field at $\mathbf{r}_0$ is,

$$\mathbf{E}_{\mathrm{t}}(\mathbf{r}_0) = \sum_{i=1}^{2N} x_i \mathbf{E}_i(\mathbf{r}_0), \tag{1}$$

where x_i is a complex number representing the amplitude and phase of one of a plane wave's two electric field components, and $\mathbf{E}_i(\mathbf{r}_0)$ is the total electric field created in the space around the nanoparticle (incorporating both incident and scattered fields) when only one plane wave component is incident with unit amplitude ($x_i = 1$). Components x_{2n-1} and x_{2n} belong to the nth plane wave. When a cold spot is created at $\mathbf{r}_0$, $\mathbf{E}_{\mathrm{t}}(\mathbf{r}_0) = 0$ and in its matrix form,

$$\mathbf{A}(\mathbf{r}_0)\mathbf{x} = 0, \tag{2}$$

where $\mathbf{A}(\mathbf{r}_0)$ is the $3 \times 2N$ matrix whose i^{th} column is the electric field $\mathbf{E}_i(\mathbf{r}_0)$, and $\mathbf{x}$ contains the complex amplitudes of all incident plane wave components

x_i,

$$\mathbf{A}(\mathbf{r}_0) = \begin{pmatrix} \uparrow & \uparrow & \uparrow & \uparrow & \uparrow \\ \mathbf{E}_1(\mathbf{r}_0) & \mathbf{E}_2(\mathbf{r}_0) & \mathbf{E}_3(\mathbf{r}_0) & \cdots & \mathbf{E}_{2N}(\mathbf{r}_0) \\ \downarrow & \downarrow & \downarrow & \downarrow & \downarrow \end{pmatrix}, \tag{3a}$$

$$\mathbf{x} = \begin{pmatrix} x_1 \\ x_2 \\ x_3 \\ \vdots \\ x_{2N} \end{pmatrix}. \tag{3b}$$

The solution (3b) of (2) is the null space of $\mathbf{A}(\mathbf{r}_0)$. Our goal is to find the electric fields $\mathbf{E}_i(\mathbf{r}_0)$ so that (2) may be solved for any cold spot position vector $\mathbf{r}_0$. The following sections use the coupled dipole model to arrive at the electric fields $\mathbf{E}_i(\mathbf{r}_0)$ when N plane waves are incident on Q particles, which we model as point scatterers.

1.1 Incident and Scattered Field

Choosing appropriate basis vectors $\hat{\mathbf{e}}_{2n-1}$ and $\hat{\mathbf{e}}_{2n}$ which are orthogonal to the nth plane wave's wavevector $\mathbf{k}_n$, an incident field made of N incident plane waves is,

$$\mathbf{E}_{\text{inc}}(\mathbf{r}) = \sum_{n=1}^{N} (x_{2n-1}\hat{\mathbf{e}}_{2n-1} + x_{2n}\hat{\mathbf{e}}_{2n})\mathrm{e}^{i\mathbf{k}_n\cdot\mathbf{r}}. \tag{4}$$

Each one of the Q total particles interacts with and scatters the electric field it sees. The superposition of each particle's scattered field, which we model as the exact field radiated by a dipole, is,

$$\mathbf{E}_{\text{sca}}(\mathbf{r}) = \frac{k_m^2}{\epsilon_0} \sum_{q=1}^{Q} \mathbf{G}(\mathbf{r}, \mathbf{r}_q)\mathbf{p}_q, \tag{5}$$

where $\mathbf{r}_q$ is the position vector of scatterer q and $\mathbf{p}_q$ is the dipole moment at its centre. The wavenumber of the medium is $k_m = \sqrt{\epsilon_m}\frac{\omega}{c}$, where ϵ_m is the medium's relative permittivity, and $\mathbf{G}(\mathbf{r}, \mathbf{r}')$ is the 3×3 dyadic Green's function evaluated at $\mathbf{r}$ with origin at $\mathbf{r}'$,

$$\mathbf{G}(\mathbf{r}, \mathbf{r}') = [\mathbf{I} - \frac{1}{k_m^2}\nabla\nabla]\frac{\mathrm{e}^{ik_m|\mathbf{r}-\mathbf{r}'|}}{4\pi|\mathbf{r}-\mathbf{r}'|}, \tag{6}$$

with $\mathbf{I}$ referring to the identity matrix, and $\nabla\nabla$ making the outer product of two nabla operators (a 3×3 matrix containing all combinations of second order partial derivatives with respect to x, y, and z). Then, the total electric field at a desired cold spot location $\mathbf{r}_0$ is found from adding all incident plane waves (4)

and scattered fields (5), evaluated at $\mathbf{r}_0$,

$$\mathbf{E}_{\mathrm{t}}(\mathbf{r}_0) = \mathbf{E}_{\mathrm{inc}}(\mathbf{r}_0) + \mathbf{E}_{\mathrm{sca}}(\mathbf{r}_0). \tag{7}$$

1.2 Coupling Effects

To account for the coupling between point scatterers, the qth dipole moment is induced both by the incident field and the sum of the fields radiated by all other dipoles,

$$\mathbf{p}_q = \boldsymbol{\alpha}_q \left(\mathbf{E}_{\mathrm{inc}}(\mathbf{r}_q) + \frac{k_m^2}{\epsilon_0} \sum_{\substack{j=1 \\ j\neq q}}^{Q} \mathbf{G}(\mathbf{r}_q, \mathbf{r}_j)\mathbf{p}_j \right). \tag{8}$$

The summation ranges from $j = 1, 2, \ldots, Q$ but excludes $j = q$ to include contributions to $\mathbf{p}_q$ from all particles other than particle q. Here, $\boldsymbol{\alpha}_q$ is the polarisability tensor associated with scatterer q. In the results presented in the main paper, we treated two spherical silver particles of 80 nm radius, $\epsilon = -8.28 + 0.78i$ illuminated by a wavelength of 500 nm in free space. The Mie scattering coefficients of these particles confirm that only the electric dipole coefficient is non-negligible, which can be written as an electric dipole polarisability α_e. While we could use the static polarisability expression for α_e, using the electric dipole Mie coefficient is more exact. The particle's polarisability tensor can be written as,

$$\boldsymbol{\alpha}_q = \begin{pmatrix} \alpha_e & 0 & 0 \\ 0 & \alpha_e & 0 \\ 0 & 0 & \alpha_e \end{pmatrix}, \tag{9}$$

$$\alpha_e = i\frac{6\pi}{k_m^3}\epsilon_0 a_1, \tag{10}$$

where $a_1 = 0.8189 - 0.3238i$ was the computed dipolar Mie coefficient for the particle considered. In order to construct $\mathbf{A}(\mathbf{r}_0)$ in (3a), we need to factorise the dipole moment of particle q, $\mathbf{p}_q$, and the dipole moments in all other particles, $\mathbf{p}_j$, so that (8) may be expressed in terms of the plane wave components x_i. This is done by absorbing coupling effects into $\boldsymbol{\alpha}_q^{\mathrm{eff}}$, an effective polarisability of particle q that takes into account multiple scattering in a self-consistent way and which multiplies the incident field only. This way, (8) is re-written as

$$\mathbf{p}_q = \boldsymbol{\alpha}_q^{\mathrm{eff}} \mathbf{E}_{\mathrm{inc}}(\mathbf{r}_q), \tag{11}$$

where unlike $\boldsymbol{\alpha}_q$, $\boldsymbol{\alpha}_q^{\mathrm{eff}}$ is not a diagonal matrix,

$$\boldsymbol{\alpha}_q^{\mathrm{eff}} = \begin{pmatrix} \alpha_{qxx}^{\mathrm{eff}} & \alpha_{qxy}^{\mathrm{eff}} & \alpha_{qxz}^{\mathrm{eff}} \\ \alpha_{qyx}^{\mathrm{eff}} & \alpha_{qyy}^{\mathrm{eff}} & \alpha_{qyz}^{\mathrm{eff}} \\ \alpha_{qzx}^{\mathrm{eff}} & \alpha_{qzy}^{\mathrm{eff}} & \alpha_{qzz}^{\mathrm{eff}} \end{pmatrix}. \tag{12}$$

The calculation of this effective polarisability matrix is explained in section 1.4.

1.3 Total Field

Combining (5), (7), and (11), the total field at some point $\mathbf{r}_0$ in space is

$$\mathbf{E}_{\mathrm{t}}(\mathbf{r}_0) = \mathbf{E}_{\mathrm{inc}}(\mathbf{r}_0) + \frac{k_m^2}{\epsilon_0}\sum_{q=1}^{Q}\mathbf{G}(\mathbf{r}_0,\mathbf{r}_q)\alpha_q^{\mathrm{eff}}\mathbf{E}_{\mathrm{inc}}(\mathbf{r}_q). \tag{13}$$

Using (4) and factorising out x_i, we can write the above as,

$$\begin{aligned}\mathbf{E}_{\mathrm{t}}(\mathbf{r}_0) = \sum_{n=1}^{N} x_{2n-1}\left[\mathrm{e}^{i\mathbf{k}_n\cdot\mathbf{r}_0}\hat{\mathbf{e}}_{2n-1} + \frac{k_m^2}{\epsilon_0}\sum_{q=1}^{Q}\mathbf{G}(\mathbf{r}_0,\mathbf{r}_q)\alpha_q^{\mathrm{eff}}\mathrm{e}^{i\mathbf{k}_n\cdot\mathbf{r}_q}\hat{\mathbf{e}}_{2n-1}\right]\\ + \sum_{n=1}^{N} x_{2n}\left[\mathrm{e}^{i\mathbf{k}_n\cdot\mathbf{r}_0}\hat{\mathbf{e}}_{2n} + \frac{k_m^2}{\epsilon_0}\sum_{q=1}^{Q}\mathbf{G}(\mathbf{r}_0,\mathbf{r}_q)\alpha_q^{\mathrm{eff}}\mathrm{e}^{i\mathbf{k}_n\cdot\mathbf{r}_q}\hat{\mathbf{e}}_{2n}\right].\end{aligned} \tag{14}$$

This master equation expresses the total field as a linear superposition of the fields developed by each plane wave component, enabling us to build the coefficient matrix $\mathbf{A}(\mathbf{r}_0)$. The square brackets in the first summation of (14) are the electric fields $\mathbf{E}_i(\mathbf{r}_0)$ for $i = 1, 3, 5, \ldots, 2N-1$, and the square brackets in the second summation are $\mathbf{E}_i(\mathbf{r}_0)$ for $i = 2, 4, 6, \ldots, 2N$, forming the columns of the matrix.

1.4 Finding Effective Polarisability

To find the elements of α_q^{eff}, we notice that (8) is itself a linear system of three equations in the dipole moment components $p_{q(x,y,z)}$ and $p_{j(x,y,z)}$. For example, rearranging (8) with particle $q = 1$ and writing it out component-wise,

$$\mathbf{p}_1 - \alpha_1\frac{k_m^2}{\epsilon_0}\sum_{j=2}^{Q}\mathbf{G}(\mathbf{r}_1,\mathbf{r}_j)\mathbf{p}_j = \alpha_1\mathbf{E}_{\mathrm{inc}}(\mathbf{r}_1) \tag{15a}$$

$$\begin{aligned}p_{1x} + b_{14}p_{2x} + b_{15}p_{2y} + b_{16}p_{2z} + b_{17}p_{3x} + b_{18}p_{3y}\\ + \cdots + b_{1(3Q)}p_{Qz} = 4\pi\epsilon_0\epsilon_m\alpha_{1x}\mathrm{E}_{\mathrm{inc}x}(\mathbf{r}_1),\end{aligned} \tag{15b}$$

$$\begin{aligned}p_{1y} + b_{24}p_{2x} + b_{25}p_{2y} + b_{26}p_{2z} + b_{27}p_{3x} + b_{28}p_{3y}\\ + \cdots + b_{2(3Q)}p_{Qz} = 4\pi\epsilon_0\epsilon_m\alpha_{1y}\mathrm{E}_{\mathrm{inc}y}(\mathbf{r}_1),\end{aligned} \tag{15c}$$

$$\begin{aligned}p_{1x} + b_{34}p_{2x} + b_{35}p_{2y} + b_{36}p_{2z} + b_{37}p_{3x} + b_{38}p_{3y}\\ + \cdots + b_{3(3Q)}p_{Qz} = 4\pi\epsilon_0\epsilon_m\alpha_{1z}\mathrm{E}_{\mathrm{inc}z}(\mathbf{r}_1),\end{aligned} \tag{15d}$$

where in the simple case of our spheres, $\alpha_{1x} = \alpha_{1y} = \alpha_{1z} = \alpha_e$. Expressed in matrix form, we have

$$\begin{pmatrix} 1 & 0 & 0 & b_{14} & b_{15} & b_{16} & \cdots & b_{1(3Q)} \\ 0 & 1 & 0 & b_{24} & b_{25} & b_{26} & \cdots & b_{2(3Q)} \\ 0 & 0 & 1 & b_{34} & b_{35} & b_{36} & \cdots & b_{3(3Q)} \end{pmatrix} \begin{pmatrix} p_{1x} \\ p_{1y} \\ p_{1z} \\ p_{2x} \\ p_{2y} \\ p_{2z} \\ \cdots \\ p_{Qz} \end{pmatrix} = 4\pi\epsilon_0\epsilon_m \begin{pmatrix} \alpha_{1x}\mathrm{E}_{\mathrm{incx}}(\mathbf{r}_1) \\ \alpha_{1y}\mathrm{E}_{\mathrm{incy}}(\mathbf{r}_1) \\ \alpha_{1z}\mathrm{E}_{\mathrm{incz}}(\mathbf{r}_1). \end{pmatrix}. \tag{16}$$

Repeating this for $q = 2, 3, \ldots, Q$ and stacking the relevant matrices together creates a solvable system of equations with a $3Q \times 3Q$ coefficient matrix $\mathbf{B}$ and a $3Q \times 1$ vector $\mathbf{c}$ on the right hand side,

$$\mathbf{B}\hat{\mathbf{p}} = \mathbf{c}. \tag{17}$$

Here, $\hat{\mathbf{p}}$ contains the components of the dipole moments induced in every particle. From (11), we find after setting $\mathbf{E}_{\mathrm{inc}}(\mathbf{r}_q) = \begin{pmatrix} 1 \\ 0 \\ 0 \end{pmatrix}$,

$$\mathbf{p}_q = \alpha_q^{\mathrm{eff}} \begin{pmatrix} 1 \\ 0 \\ 0 \end{pmatrix} = \begin{pmatrix} \alpha_{qxx}^{\mathrm{eff}} \\ \alpha_{qyx}^{\mathrm{eff}} \\ \alpha_{qzx}^{\mathrm{eff}} \end{pmatrix}, \tag{18}$$

which means that the solution of (18) when $\mathbf{E}_{\mathrm{inc}}(\mathbf{r}_q) = \begin{pmatrix} 1 \\ 0 \\ 0 \end{pmatrix}$ for all q contains the first column of every particle's effective polarisability. The remaining two columns may be found from solving (18) with $\mathbf{E}_{\mathrm{inc}}(\mathbf{r}_q) = \begin{pmatrix} 0 \\ 1 \\ 0 \end{pmatrix}$ and $\mathbf{E}_{\mathrm{inc}}(\mathbf{r}_q) = \begin{pmatrix} 0 \\ 0 \\ 1 \end{pmatrix}$.

1.5 Finding $\mathbf{A}(\mathbf{r}_0)$ for an Arbitrary Particle

Since the columns of $\mathbf{A}(\mathbf{r}_0)$ are the electric fields directly set up by x_i, putting together the system matrix for an arbitrarily shaped particle is a straightforward procedure. Conduct a total of $2N$ simulations, one pair per plane wave. In each simulation pair, a single plane wave illuminates with polarisations $\mathbf{E}_n(\mathbf{r} = \mathbf{0}) = \hat{\mathbf{e}}_{2n-1}$ and $\mathbf{E}_n(\mathbf{r} = \mathbf{0}) = \hat{\mathbf{e}}_{2n}$, while the electric field is probed at $\mathbf{r}_0$, such that one

simulation returns one column of $\mathbf{A}(\mathbf{r}_0)$. Calculating the null space of $\mathbf{A}(\mathbf{r}_0)$, the electric fields from each simulation can be scaled by x_i and superimposed to reveal the manufactured cold spot.

2 Equation 4 in Main Paper

A smooth function of $\mathbf{r}$, like $\mathbf{E}_\mathrm{t}(\mathbf{r})$, is almost linear over small distances. We can make a first-order approximation of the total electric field to capture its behaviour in the vicinity of the cold spot as follows,

$$\tilde{\mathbf{E}}_\mathrm{t}(\mathbf{r}) = \mathbf{J}\mathbf{v}, \tag{19}$$

where $\mathbf{J}$ is the Jacobian of the exact total electric field (7) evaluated at $\mathbf{r}_0$,

$$\mathbf{J} = \begin{pmatrix} \frac{\partial \mathrm{E}_{\mathrm{t}x}}{\partial x}\Big|_{\mathbf{r}_0} & \frac{\partial \mathrm{E}_{\mathrm{t}x}}{\partial y}\Big|_{\mathbf{r}_0} & \frac{\partial \mathrm{E}_{\mathrm{t}x}}{\partial z}\Big|_{\mathbf{r}_0} \\ \frac{\partial \mathrm{E}_{\mathrm{t}y}}{\partial x}\Big|_{\mathbf{r}_0} & \frac{\partial \mathrm{E}_{\mathrm{t}y}}{\partial y}\Big|_{\mathbf{r}_0} & \frac{\partial \mathrm{E}_{\mathrm{t}y}}{\partial z}\Big|_{\mathbf{r}_0} \\ \frac{\partial \mathrm{E}_{\mathrm{t}z}}{\partial x}\Big|_{\mathbf{r}_0} & \frac{\partial \mathrm{E}_{\mathrm{t}z}}{\partial y}\Big|_{\mathbf{r}_0} & \frac{\partial \mathrm{E}_{\mathrm{t}z}}{\partial z}\Big|_{\mathbf{r}_0} \end{pmatrix}, \tag{20}$$

and $\mathbf{v} = \mathbf{r} - \mathbf{r}_0$. With this approximation, the square of the magnitude of the total field is,

$$|\tilde{\mathbf{E}}_\mathrm{t}(\mathbf{r})|^2 = \tilde{\mathbf{E}}_\mathrm{t}(\mathbf{r}) \cdot \tilde{\mathbf{E}}^*_\mathrm{t}(\mathbf{r}) = \mathbf{J}\mathbf{v} \cdot \mathbf{J}^*\mathbf{v} = (\mathbf{J}\mathbf{v})^T \mathbf{J}^*\mathbf{v}. \tag{21}$$

Using $(AB)^T = B^T A^T$,

$$|\tilde{\mathbf{E}}_\mathrm{t}(\mathbf{r})|^2 = \mathbf{v}^T \mathbf{J}^T \mathbf{J}^* \mathbf{v}. \tag{22}$$

The electric field intensity is a real quantity. Even though $\mathbf{J}^T\mathbf{J}^*$ is complex-valued, we find that $\mathbf{v}^T \mathrm{Im}\{\mathbf{J}^T\mathbf{J}^*\}\mathbf{v} = 0$ for all $\mathbf{v}$ and therefore,

$$\mathbf{v}^T \mathbf{J}^T \mathbf{J}^* \mathbf{v} = \mathbf{v}^T \mathrm{Re}\{\mathbf{J}^T\mathbf{J}^*\}\mathbf{v}. \tag{23}$$

Electric field intensity cannot be negative. Because the cold spot is a point of zero intensity by definition, $|\tilde{\mathbf{E}}_\mathrm{t}(\mathbf{r})|^2$ must, in general, increase in all directions away from $\mathbf{r}_0$ meaning $\mathrm{Re}\{\mathbf{J}^T\mathbf{J}^*\}$ is a positive-definite matrix. The right hand side of (23), when set to a constant value C, satisfies the general equation for an ellipsoid whose semi-axes directions are given by the eigenvectors of $\mathrm{Re}\{\mathbf{J}^T\mathbf{J}^*\}$. The ellipsoid semi-axis lengths are the inverse square root of the eigenvalues of $\mathrm{Re}\{\mathbf{J}^T\mathbf{J}^*\}$ when the ellipsoid surface is defined at $|\tilde{\mathbf{E}}_\mathrm{t}(\mathbf{r})|^2 = 1$. If we adopt co-ordinate axes (x', y', z') which are aligned to the ellipsoid principle axes, and normalise $|\tilde{\mathbf{E}}_\mathrm{t}(\mathbf{r})|^2$ by $|\mathbf{x}|^2$ then the eigenvalues of $\frac{1}{|\mathbf{x}|^2}\mathrm{Re}\{\mathbf{J}^T\mathbf{J}^*\}$ satisfy,

$$\lambda_1(x' - x'_0)^2 + \lambda_2(y' - y'_0)^2 + \lambda_3(z' - z'_0)^2 = \frac{|\tilde{\mathbf{E}}_\mathrm{t}(\mathbf{r})|^2}{|\mathbf{x}|^2}. \tag{24}$$

This means λ_1, λ_2, and λ_3, measured in nm^{-2}, can inform us of how narrow the cold spot ellipsoid is along its principle axes. A lower-dimensional zero like

a cold line or a cold plane could be thought of as a special case where the ellipsoid is infinitely elongated in one or two directions. This way, one or two of the ellipsoid's eigenvalues would be zero. As long as all three eigenvalues are non-zero, then, we can be confident that the cold spot has a three-dimensional confinement.

3 Background Refractive Index Sensitivity

To make a cold spot near a nanoantenna relies on prior knowledge of the way the nanoantenna responds to each linear component of two incident plane waves. This depends on the nanoantenna's position, geometry, and material, as well as the refractive index of the surrounding medium. Here, we test the response of a cold spot, designed in free space, to an increase in background refractive index. This emulates the effect that organic particles like viruses can have on optical systems (the basis for many sensing methods). A cold spot is created at $\mathbf{r}_0$ exactly in the nanogap of the two particles, modelled as point-dipoles, in Fig. 2 of the main paper. Assuming these particles are silver ($\epsilon_{\mathrm{Ag}} = -8.28 + 0.78i$ at a free space wavelength of 500 nm) and suspended in free space, the nullspace of $\mathbf{A}(\mathbf{r}_0)$ is found and the cold spot-creating linear components of the two plane waves, $\mathbf{E}_\mathrm{a}$ and $\mathbf{E}_\mathrm{b}$, are obtained. Keeping this illumination fixed, the refractive index of the background is increased from 1 to 1.5 and the electric field magnitude in $\mathbf{r}_0$ is measured and plotted in arbitrary units in Fig. S1 (assuming $|\mathbf{x}|^2 = |\mathbf{E}_\mathrm{a}|^2 + |\mathbf{E}_\mathrm{b}|^2 = 1$ arb. u.^{2}). By altering the background index, the Mie coefficients and polarisability of the particles change in addition to the particle coupling and the phase of the incident field exciting the electric dipoles. The cold spot is sensitive even to very small changes in the background refractive index; an increase of as little as $+0.05$ causes a significant rise in field magnitude in the cold spot.

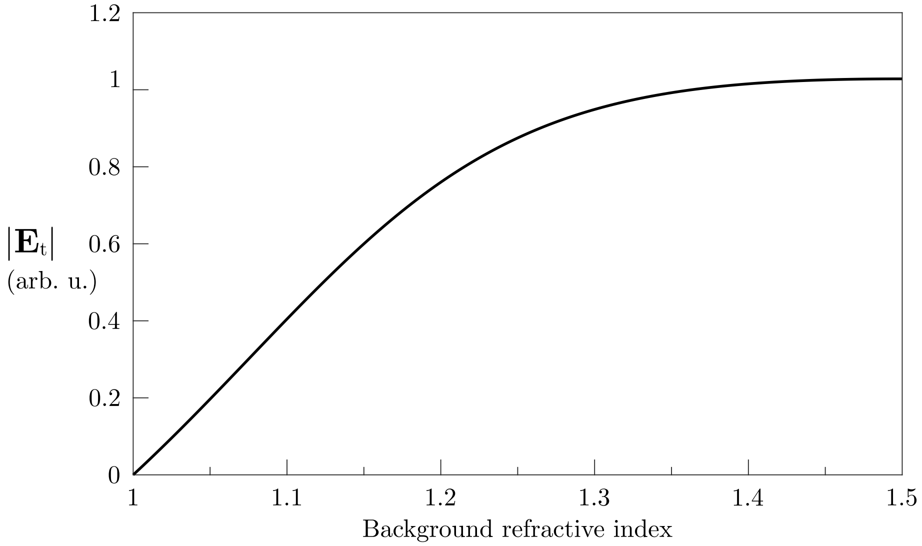

Figure S1: Response of a cold spot to changes in the refractive index of the background medium, in the two-dipole analytical model presented in the main paper. The electric field magnitude is measured only in the cold spot position, exactly between the two dipoles, in arbitrary units, assuming the incident intensity $|\mathbf{E}_\mathrm{a}|^2 + |\mathbf{E}_\mathrm{b}|^2 = 1$ arb. u.^{2}.
